# Prevention of hypoglycemia-induced neuronal death by minocycline

**DOI:** 10.1186/1742-2094-9-225

**Published:** 2012-09-22

**Authors:** Seok Joon Won, Jin Hee Kim, Byung Hoon Yoo, Min Sohn, Tiina M Kauppinen, Man-Seong Park, Hyung-Joo Kwon, Jialing Liu, Sang Won Suh

**Affiliations:** 1 Department of Neurology, University of California San Francisco and Veterans Affairs Medical Center, San Francisco, CA, 94121, USA; 2 Department of Neurological Surgery, University of California San Francisco and Veterans Affairs Medical Center, San Francisco, CA, 94121, USA; 3 Departments of Anesthesiology, Inje Paik Hospital, Inje University, School of Medicine, Seoul, 139-707, Korea; 4 Department of Nursing, Inha University, Incheon, 402-751, Korea; 5 Department of Microbiology, Hallym University, School of Medicine, Chuncheon, 200-702, Korea; 6 Department of Physiology, Hallym University, School of Medicine, Chuncheon, 200-702, Korea

**Keywords:** Hypoglycemia, Minocycline, Neuronal death, Microglia

## Abstract

Diabetic patients who attempt strict management of blood glucose levels frequently experience hypoglycemia. Severe and prolonged hypoglycemia causes neuronal death and cognitive impairment. There is no effective tool for prevention of these unwanted clinical sequelae. Minocycline, a second-generation tetracycline derivative, has been recognized as an anti-inflammatory and neuroprotective agent in several animal models such as stroke and traumatic brain injury. In the present study, we tested whether minocycline also has protective effects on hypoglycemia-induced neuronal death and cognitive impairment. To test our hypothesis we used an animal model of insulin-induced acute hypoglycemia. Minocycline was injected intraperitoneally at 6 hours after hypoglycemia/glucose reperfusion and injected once per day for the following 1 week. Histological evaluation for neuronal death and microglial activation was performed from 1 day to 1 week after hypoglycemia. Cognitive evaluation was conducted 6 weeks after hypoglycemia. Microglial activation began to be evident in the hippocampal area at 1 day after hypoglycemia and persisted for 1 week. Minocycline injection significantly reduced hypoglycemia-induced microglial activation and myeloperoxidase (MPO) immunoreactivity. Neuronal death was significantly reduced by minocycline treatment when evaluated at 1 week after hypoglycemia. Hypoglycemia-induced cognitive impairment is also significantly prevented by the same minocycline regimen when subjects were evaluated at 6 weeks after hypoglycemia. Therefore, these results suggest that delayed treatment (6 hours post-insult) with minocycline protects against microglial activation, neuronal death and cognitive impairment caused by severe hypoglycemia. The present study suggests that minocycline has therapeutic potential to prevent hypoglycemia-induced brain injury in diabetic patients.

## Introduction

Hypoglycemia occurs in type 1 and type 2 diabetic patients who attempt strict management of their blood glucose levels with insulin or other glucose-lowering drugs [1-3]. Hypoglycemia causes recurrent morbidity in patients, and sometimes results in mortality [4,5]. Frequent low blood glucose levels in type 1 diabetic patients can lead to the development of hypoglycemia unawareness, which desensitizes patients to symptoms of low blood glucose [6]. Hypoglycemia unawareness may lead to prolonged nocturnal hypoglycemia, causing convulsions and coma, and resulting in sudden death [7]. Severe hypoglycemia, most commonly encountered in diabetic patients who unintentionally self-administer insulin at supratherapeutic doses, can cause potential complications such as irritability, impaired concentration, focal neurological deficits, confusion, drowsiness, coma, seizure, and neuronal death [8]. Under the most severe conditions, hypoglycemic neuronal death occurs in CA1, subiculum and dentate gyrus of the hippocampus, neurons in cortical layers II and III of the cerebral cortex, and the dorsolateral striatum. Hippocampal neurons are particularly important for learning and memory, and impairment of cognitive abilities is the most common sequelae of hypoglycemic coma [9]. We and other groups have reported that hypoglycemia-induced neuronal death is not a simple result of energy failure resulting from low glucose, but the result of activating cell death-related signaling pathways such as glutamate receptor activation, reactive oxygen species (ROS) production and extracellular zinc release [10-12].

Microglia are the major innate immune cells in the brain. Once microglia are activated by neurological damage or systemic inflammatory events, activated microglia release neurotoxic substances such as nitric oxide, ROS, cytokines, and chemokines, and undergo morphological changes from a ramified to an amoeboid shape [13,14]. As well, the recruitment of various types of peripherally derived inflammatory cells such as neutrophils, T cells, and macrophages can affect brain inflammation following neurological damage [15]. Inhibition of inflammation increases the rates of neuronal survival and improves neurological deficits in several animal models of neurological injury [16,17]. Although the exact circumstances under which microglial activation is harmful or beneficial are still controversial [15], some evidence indicates that early phase inflammation caused by acute brain injury can contribute to neuronal death. In a previous study, we showed that hypoglycemic injury-induced microglial activation is affected by body temperature [18]. However, it is unknown whether microglial activation is a major contributing factor in hypoglycemia-induced neuronal death.

Minocycline is a second-generation semi-synthetic tetracycline derivative that possesses improved tissue penetration into the cerebrospinal fluid and the central nervous system, compared to earlier forms. Beside the anti-microbial effect, minocycline has anti-inflammatory and anti-apoptotic effects. It has been shown that minocycline has neuroprotective effects in animal models of neurodegenerative disease including Parkinson’s [19], Huntington’s [20] and Alzheimer’s disease [21], as well as several animal models of neurological disease such as global ischemia [22], focal cerebral ischemia [23] and spinal cord injury [24]. These effects are thought to arise through the inhibition of microglial activation, inducible nitric oxide synthase, COX-2 expression or modulation of cytokine expression. In the present study, we tested whether delayed treatment with minocycline could also reduce microglial activation, neuronal death and cognitive impairment induced by severe hypoglycemia.

## Materials and methods

### Animal surgery and insulin-induced hypoglycemia

This study was approved by the San Francisco Veterans Affairs Medical Center animal studies committee. Hypoglycemia was induced by injection of regular human insulin as described by Auer *et al*. [25] with minor modifications [26]. Briefly, male Sprague–Dawley rats (Charles River Laboratories, Gilroy, CA, USA) weighing between 250 and 350 g were fasted overnight but allowed free access to water. Hypoglycemia was induced by intraperitoneal injection of 10 U/kg of regular insulin (Novolin-R, Novo Nordisk, Clayton, NC, USA). The rats were anesthetized with 2% isoflurane in a 75:25 mixture of nitrous oxide and oxygen. After tracheal intubation, controlled ventilation (Harvard Apparatus, South Natick, MA, USA) was initiated and anesthesia was maintained with 1% isoflurane. A 26-gauge polyvinyl catheter was introduced into the femoral artery for continuous arterial blood pressure monitoring and blood sampling, and another 26-gauge polyvinyl catheter was placed in the femoral vein for glucose infusion. Cranial trephinations were performed over the bilateral parietal cortex for electroencephalogram (EEG) monitoring (BIOPAC system Inc, Santa Barbara, CA, USA). Two monopolar electrodes were placed beneath the dura and another reference electrode was inserted into neck muscle. Body temperature was maintained at 36.5 to 37.5°C using a warming pad during surgery. Hypoglycemia was terminated after 30 minutes of iso-EEG to generate a reproducible brain injury of moderate severity [25]. During the entire period of EEG isoelectricity, mean arterial blood pressure was maintained between 120 and 180 mmHg by adjusting isoflurane concentration. Blood pressure and blood glucose levels in animals belonging to vehicle treated and minocycline treated group showed no difference throughout the experiment (Table 1 and 2). Bradycardia and respiratory track secretion was prevented by administration of 1 mg/kg of atropine. Hypoglycemia was terminated by administration of 0.2 ml of 50% glucose via the femoral vein, followed by continuous intravenous infusion of 1:1 solution of 50% glucose and Krebs-Henseleit buffer at a rate of 1.5 ml/h for 3 hours. Rats were recovered from anesthesia after two more hours of intravenous glucose infusion. In sham hypoglycemia, rats were fasted overnight and received the same dose of insulin, but were then maintained in a normoglycemic state by intravenous administration of 25% glucose (1.5 ml/3 h).

**Table 1:** Arterial blood glucose change before, during and after hypoglycemia (mM)

	**Before hypoglycemia**	**Iso-EEG**	**After glucose reperfusion**
Vehicle group (n = 6)	4.02 ± 0.13	0.40 ± 0.03	7.93 ± 0.16
Minocycline group (n = 6)	4.08 ± 0.12	0.38 ± 0.01	7.90 ± 0.23

Iso-EEG, isoelectric electroencephalogram.

**Table 2:** Arterial blood pressure change before, during and after hypoglycemia (mmHg)

	**Before hypoglycemia**	**Iso-EEG**	**After glucose reperfusion**
Vehicle group (n = 6)	103.34 ± 1.88	185.07 ± 3.29	107.95 ± 3.85
Minocycline group (n = 6)	101.91 ± 1.79	184.29 ± 3.13	106.44 ± 4.27

Iso-EEG, isoelectric electroencephalogram.

### Minocycline injection

After hypoglycemic surgery or sham operation, rats in each group were randomly assigned to two subgroups. Minocycline (Sigma, St Louis, MO, USA) dissolved in 0.1 M phosphate buffer, pH 7.4 (50 mg/kg) or vehicle was administered intraperitoneally once daily beginning 6 hours after glucose reperfusion for a duration of 1 week (total of seven times).

### Immunohistochemistry

At the indicated time points, rats were anesthetized and transcardially perfused with 200 ml of 0.9% saline followed by 200 ml of 4% formaldehyde (FA). The harvested brains were post-fixed for 24 hours and immersed in 20% sucrose. Free-floating coronal sections (40 μm thickness) were incubated with blocking buffer (10% goat serum and 0.1% Triton X-100 in 0.1 M PB) for 30 minutes at room temperature. The sections were then incubated with mouse anti-rat CD11b (1 μg/ml; Serotec, Raleigh, NC, USA), mouse anti-NeuN (1 μg/ml; Millipore, Billerica, MA, USA) or rabbit anti-myeloperoxidase (1:200; Thermo scientific, Fremont, CA, USA) overnight. After washing, the sections were incubated with biotinylated anti-mouse or anti-rabbit IgG secondary antibody (20 μg/ml; GE Healthcare, UK) for 2 hours. Sections were processed with ABC reagents using a Vector ABC kit (Vector laboratories, Burlingame, CA, USA). The horseradish peroxidase reaction was detected with diaminobenzidine and H_2_O_2_. For immunofluorescence staining, the sections were incubated with Alexa Fluor 488-conjugated goat anti-mouse IgG secondary antibody (Invitrogen, Carlsbad, CA, USA) at a dilution of 1:500 for 2 hours at room temperature. Negative controls performed with secondary antibody alone showed no staining.

### Assessment of neuronal survival

At 7 days and 8 weeks after hypoglycemia, rats were killed and their brains were harvested. The sections were immunostained with anti-NeuN. The number of NeuN-stained CA1 neurons was determined in every twelfth coronal section spanning the septal hippocampus (Bregma level −1.8 to −3.8) using unbiased stereology (Stereo Investigator, MicroBrightField, Williston, VT, USA). A counting frame (15 × 15 × 20 μm) was placed at the intersection of a matrix (200 × 200 μm) randomly superimposed onto the region of interest by the program.

### Assessment of neuronal death

For identifying degenerating neurons in the hippocampus, Fluoro-Jade B (FJB) staining was performed as described previously [27]. Briefly, sections were immersed in a basic alcohol solution for 5 minutes and 0.06% KMnO_4_ for 15 minutes, and then the sections were incubated in 0.0004% FJB (Histo-Chem, Jefferson, AR, USA) for 20 minutes. The slides were washed in distilled water, and then dried. To quantify neuronal death, every twelfth coronal section spanning the septal hippocampus (Bregma level −1.8 to −3.8) was analyzed from each animal. A blinded observer to the minocycline treatment condition counted the number of FJB positive (+) neurons in the hippocampal CA1 from both hemispheres. Mean counts of FJB (+) neurons from each region were used for statistical analysis.

### Immunohistochemistry for evaluation of microglial activation

CD11b immunohistochemistry was performed to determine the level of microglial activation. Microglial activation was evaluated by a blinded observer and scored based on cell number, morphology and CD11 expression intensity (Table 3) as modified from a previously published method [28]. Three sections from each animal were evaluated.

**Table 3:** Scoring for microglial activation

**Score**	**Cell number (cell number per 200 μm × 200 μm)**	**Morphology (% with amoeboid morphology)**	**CD11b intensity**
0	0	0%	No expression
1	1-20	1-45%	Weak
2	21-40	46-90%	Average
3	> 40	> 90%	High

Microglia were identified by CD11b immunostaining. Scoring with three criteria were combined to yield an aggregate microglial activation score (0 to 9).

### Assessment of infiltrated neutrophils

At 3 days after hypoglycemia, rats were killed and their brains were harvested. The sections were immunostained with anti-myeloperoxidase (anti-MPO). A blinded observer counted the number of MPO-immunopositive cells in the cortex and hippocampus of both hemispheres. Three sections from each animal were evaluated.

### Behavioral assessments

Six weeks following the sham operation or hypoglycemia, rats were first tested in the novel open field to evaluate the effects of the delayed treatment with minocycline on locomotor activity and exploratory behavior following hypoglycemia. They were then assessed by the Barnes maze test to evaluate spatial learning and memory [29]. In the open field test, rats were placed in a brightly lit, square plexiglas enclosure (40 × 40 inches) surrounded by automated infrared photocells interfaced with a computer (Hamilton Kinder, San Diego, CA, USA) to record the data. Beam-breaks generated by movement were monitored, allowing measurements of the number of movements, distance moved, and time spent in the enclosure or zones. On each of three consecutive days, open field activity was recorded for 10 minutes after an initial 1-minute adaptation period. For analysis of the exploratory behavior, the arena was divided on a zone map consisting of a center region (15 × 15 inches^2^), four corner regions of 7.5 × 7.5 inches each, and a peripheral region (the remaining area). In the Barnes maze, a black acrylic escape tunnel was placed under one of the holes on a circular platform (120 cm in diameter) with 18 holes (10 cm in diameter per hole) along the platform perimeter (Hamilton Kinder). The platform was elevated 60 cm above the floor. Rats from each treatment group were randomly assigned to locate the escape tunnel from one of the three pre-determined locations to rule out spatial preference. Mildly noxious stimuli, blowing fans and 500 LUX of bright light, were used to increase the incentive in finding the escape tunnel. The Noldus EthoVision video tracking system (Noldus, Leesburg, VA, USA) was used to record and analyze the data. Rats were trained to locate the escape tunnel in two successive daily sessions for 5 days (three trials per session, three minutes per trial) with a one-hour intersession interval from different counterbalanced starting positions.

### Statistical analysis

Data are shown as means ± standard error of the mean (SEM). Statistical significance was assessed by analysis of variance (ANOVA) and post hoc testing was accomplished using the Bonferroni test. *P*-values less than 0.05 were considered statistically significant. Behavioral data were evaluated by two-way repeated measures ANOVA (RANOVA) followed by post hoc pair-wise comparisons using the Bonferroni test. Microglial activation data were assessed by Kruskal-Wallis non-parametric one-way ANOVA followed by Dunn’s test for multiple group comparison.

## Results

### Hypoglycemic brain injury induces microglial activation in the brain

**Figure 1:**
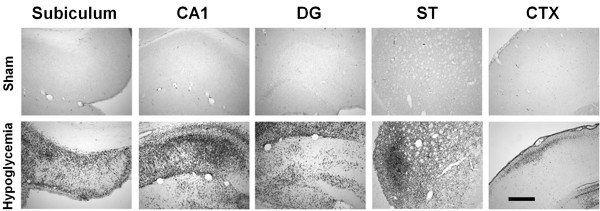
**Hypoglycemia induces microglial activation in the brain.** Light microscopy images show microglial activation after hypoglycemia. In the sham operated animal, CD11b-positive cells are negligible in all brain areas (Sham). However, a substantial degree of microglial activation is detected in the hippocampus (Subiculum, CA1, and dentate gyrus (DG)), striatum (ST) and cerebral cortex (CTX) 5 days after hypoglycemia. This histological finding suggests that the inflammatory response is involved in hypoglycemia-induced neuronal injury. Scale bar = 200 μm.

To characterize microglial activation following hypoglycemia, we performed immunohistochemistry with anti-CD11b, which detects activated microglia, at 5 days after hypoglycemia. A substantial degree of microglial activation was detected in the hippocampus, striatum and cerebral cortex 5 days after hypoglycemia (Figure 1). This histological finding suggests that the inflammatory response is involved in hypoglycemia-induced neuronal death.

**Figure 2:**
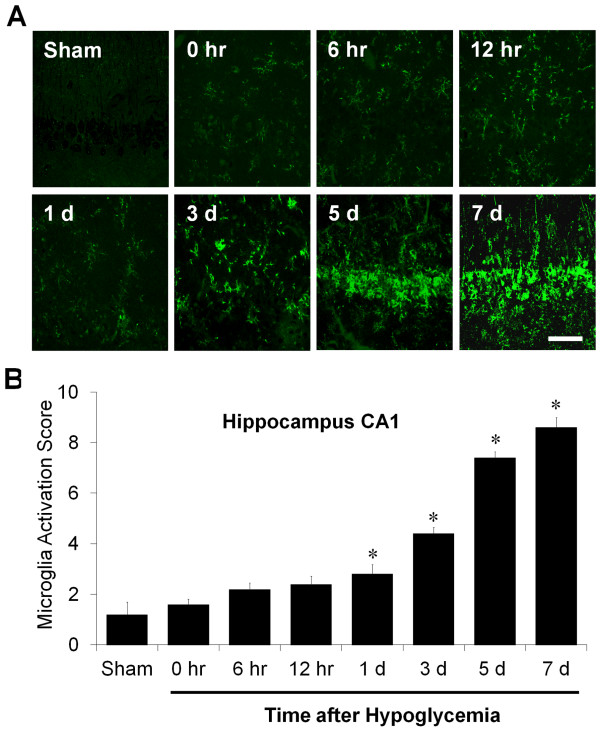
**Time course of microglial activation in the hippocampal CA1 region after hypoglycemic brain injury. (A)** Representative images of microglial activation on the brain sections at indicated time points after hypoglycemic brain injury. Activated microglia were immunostained with anti-CD11b. Scale bar = 40 μm. **(B)** Quantification of microglial activation in the hippocampal CA1 area of rats after hypoglycemic brain injury. Microglial activation is scored as described in the Methods and Materials. Data are mean ± standard error of the mean (SEM); ^#^*P <* 0.05 compared with sham-operated animals, *P < 0.05 compared with injured animals treated with saline (n = 5).

To evaluate the time-dependent activation of microglia in the hippocampal area following hypoglycemia injury, immunohistochemistry for CD11b was performed at the designated time points. There was no immunoreactive signal in the CA1 of the hippocampal area in sham-operated animals. Microglial activation was detected starting 1 day after the hypoglycemic insult and gradually increased. At days 5 and 7 after hypoglycemia, robustly activated microglia were detected in the CA1 area of the hippocampus (Figure 2).

### Minocycline inhibits microglial activation after hypoglycemia

**Figure 3:**
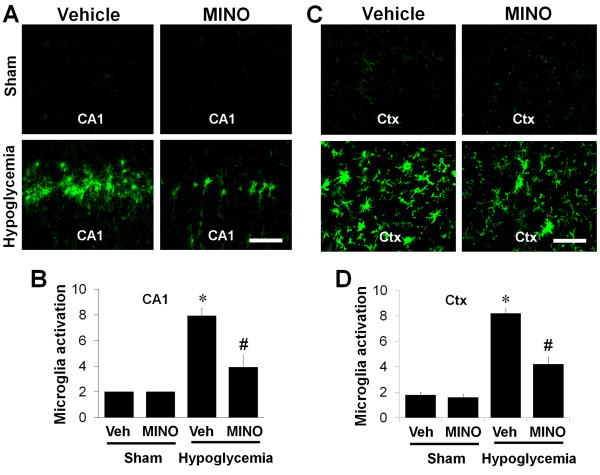
**Minocycline reduces microglial activation after hypoglycemic brain injury.** Images show that microglial activation is reduced in the hippocampal CA1 area (CA1) **(A)** and in the perirhinal cortex area (Ctx) **(C)** by minocycline treatment after hypoglycemia. Rats received intraperitoneal injections of saline or minocycline (MINO) at 6 hours after hypoglycemia. Injections were continued once per day for 6 days. Microglial activation was evaluated 7 days after hypoglycemia or sham operation. Brain sections from sham-operated (Sham) or hypoglycemic-injured (Hypoglycemia) rats were immunostained with anti-CD11b. Note that activated microglial cells, which showed amoeboid morphology with enlarged soma and thickened processes, were reduced by the delayed treatment with minocycline. Scale bar = 40 μm. Quantification of microglial activation in the hippocampal CA1 area (CA1) **(B)** and cerebral cortex (Ctx) **(D)** of rats receiving minocycline (MINO) or vehicle (Veh) after hypoglycemic brain injury. Microglial activation is scored as described in the Methods and Materials. Data are mean ± standard error of the mean (SEM); *P < 0.05 compared with sham-operated animals, ^#^*P <* 0.05 compared with injured animals treated with saline (Veh) (n = 6).

In Figure 2, we show that activation of microglia appears at 24 hours after hypoglycemia in the hippocampus. In order to test the ability of minocycline to directly prevent microglial activation, (as opposed to prevention of microglial activation via direct neuroprotection and the inhibition of cell death signaling that might occur if treatment was administered immediately after hypoglycemia induced-brain damage) we injected minocycline at 6 hours after glucose reperfusion and then once daily for 1 week. Compared with the sham-operated group, hypoglycemia increased microglial activation in the hippocampal and in the cortical area; however, the delayed treatment with minocycline reduced this activation. The number of amoeboid cells, number of CD11b-positive cells and intensity of the CD11b fluorescent signal was significantly reduced by minocycline treatment (Figure 3).

### Minocycline reduces hippocampal neuronal death after hypoglycemia

**Figure 4:**
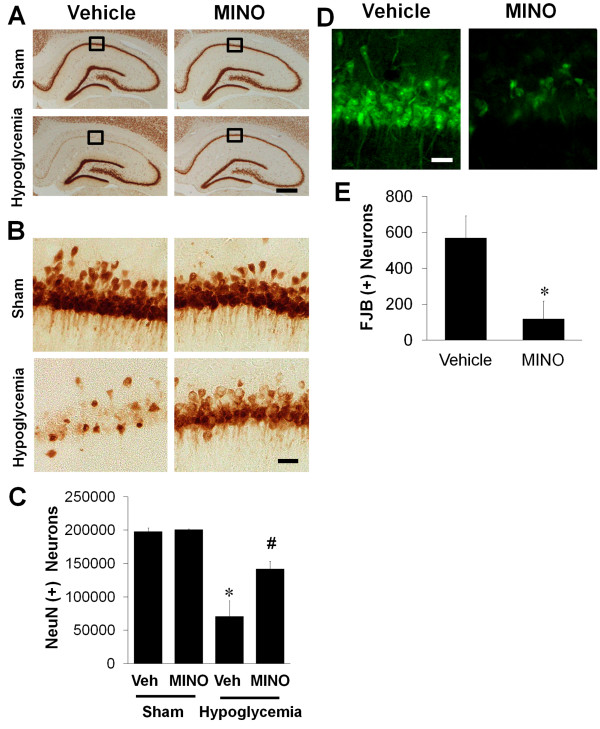
**Minocycline prevents hypoglycemia-induced neuronal death in the hippocampal CA1 area. (A)** Representative low-magnification images of surviving neurons in the hippocampus by minocycline treatment after hypoglycemia. Brain sections from sham-operated (Sham) or hypoglycemia experienced (Hypoglycemia) rats were immunostained with anti-NeuN. Scale bar = 500 μm. **(B)** Representative high magnification of surviving neurons in hippocampal CA1 area by minocycline treatment after hypoglycemia. Note that hypoglycemia-induced hippocampal CA1 neuronal death was protected by the delayed minocycline treatment. Scale bar = 40 μm. **(C)** Quantification of surviving neurons in the hippocampal CA1 area of rats treated by minocycline (MINO) or vehicle (Veh) after hypoglycemia. Data are mean ± standard error of the mean (SEM); *P < 0.05 compared with sham-operated animals, ^#^*P <* 0.05 compared with hypoglycemia animals treated with saline (Veh) (n = 6). **(D)** Representative images of degenerating cells in the hippocampal CA1 area following minocycline (MINO) or saline (Vehicle) treatment after hypoglycemia. Brain sections were stained by Fluoro-Jade B (FJB). Note that minocycline reduced the number of FJB (+) cells in the hippocampal CA1 area after hypoglycemia. Scale bar = 40 μm. **(E)** Quantification of Fluoro-Jade B (+) cells in the hippocampal CA1 area from rats treated with minocycline after hypoglycemia. Data are mean ± SEM; *P < 0.05 compared with hypoglycemia-subjected rats treated with saline (n = 6).

Minocycline has been described to act as a neuroprotective agent in animal models of cerebral ischemia and spinal cord injury. In the present study, we injected minocycline at 6 hours after reperfusion to investigate its neuroprotective effect after hypoglycemia. Immunohistochemistry with anti-NeuN was performed to estimate the survival of CA1 neurons in the hippocampus. At 7 days after hypoglycemia, the NeuN-positive CA1 layer was almost absent, compared with sham-operated animals. But the post-treatment with minocycline spared the NeuN-positive cells in the CA1 layer (Figure 4A). Under high-magnification, the number of NeuN-positive CA1 neurons was seen to be increased by minocycline treatment (Figure 4B). As we counted the number of live neurons, NeuN-positive neurons in the CA1 area decreased in rats who experienced hypoglycemia by about 80% compared to sham-operated rats. However, the delayed treatment with minocycline significantly increased the number of NeuN-positive neurons (Figure 4C). As observed in anti-NeuN immunohistochemistry, minocycline reduced the degenerating cells labeled by FJB staining cells compared with hypoglycemic-injured animals (Figure 4D,E).

### Minocycline reduces MPO immunoreactivity induced by hypoglycemia

**Figure 5:**
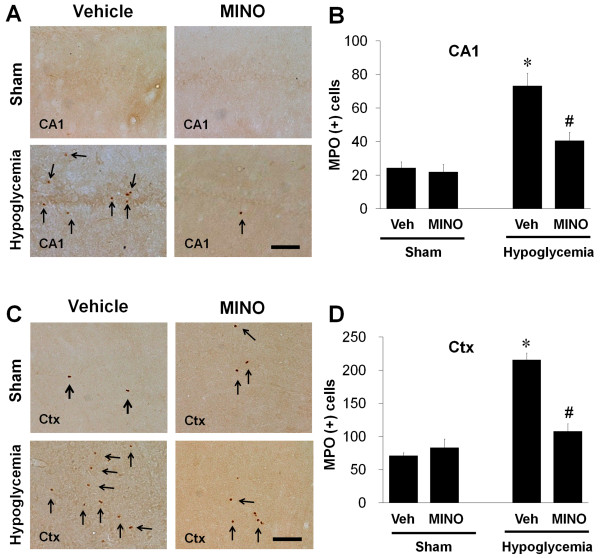
**Minocycline reduces myeloperoxidase (MPO) immunoreactivity induced by hypoglycemia in the hippocampus.** Representative images of reduction in MPO immunoreactive cells in the hippocampal CA1 area (CA1) **(A)** and in the perirhinal cortex (Ctx) **(C)** by minocycline treatment after hypoglycemia. Note that hypoglycemia-induced MPO-positive infiltrating cells were reduced by the delayed minocycline treatment. Scale bar = 40 μm. Quantification of MPO-positive cells in the hippocampus (CA1) **(B)** and in the cortex (Ctx) **(D )** of rats treated with minocycline (MINO) or saline (Vehicle) after hypoglycemia. The number of MPO (+) cells was counted under microscope as described in Methods and Materials. Data are mean ± standard error of the mean (SEM); *P < 0.05 compared with sham-operated animals, ^#^*P <* 0.05 compared with hypoglycemia animals treated with saline (Vehicle) (n = 7).

It has been reported that infiltrating neutrophils contribute to neurological brain damage [30,31]. To test whether infiltrating neutrophils are observed in hypoglycemic brain injury and whether post-treatment with minocycline can reduce the presence of infiltrating neutrophils, we performed immunohistochemistry with anti-MPO to observe the degree of neutrophil infiltration in the post-hypoglycemic hippocampus. In saline- or minocycline-treated sham animals, MPO-positive cells were sparse in the hippocampus. Hypoglycemic injury increased the number of infiltrating neutrophils in the CA1 pyramidal layer and in the cortex layer II/III, an area that is vulnerable to hypoglycemia. However, minocycline reduced neutrophilic infiltration in the hippocampal formation, mainly in the CA1 layer and in the cortex layer II/III (Figure 5).

### Minocycline reduces motor hyperactivity caused by hypoglycemia

**Figure 6:**
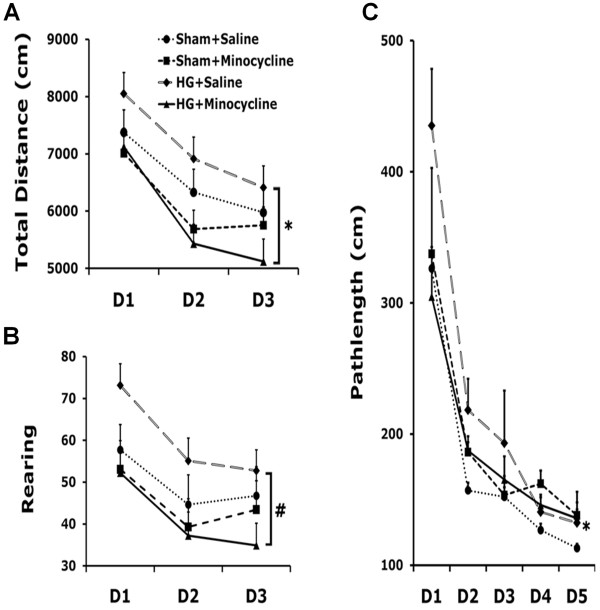
**Minocycline reduces motor hyperactivity and cognitive impairment induced by hypoglycemia.** Six weeks after induction of hypoglycemia, rats were subjected to a 3-day novel open field test followed by the Barnes maze test. Four groups of animals were divided for behavioral testing as follows: sham + Vehicle; sham + minocycline (MINO); hypoglycemia + Vehicle; and hypoglycemia + minocycline (MINO) groups. Minocycline reduced overall hyperactivity induced by hypoglycemia in both horizontal and vertical dimensions as shown in total path length **(A)** and rearing events **(B)**. Minocycline also improved the performance of rats with hypoglycemia in the Barnes maze test over 5 days of training (**C**). *P < 0.05, ^#^*P <* 0.01 comparing HG + Vehicle and HG + MINO groups.

To investigate whether hypoglycemia or minocycline affects locomotor activity, rats were subjected to an open field test at 6 weeks after hypoglycemia. All groups were able to habituate over 3 days of testing in the novel open field. Rats who experienced hypoglycemia showed increased activity both horizontally and vertically (Figure 6A,B; total path length: *P <* 0.0001; rearing: *P <* 0.0001). Minocycline reduced hypoglycemia-induced hyperactivity both horizontally (total path length; *P <* 0.05) and vertically (rearing; *P <* 0.01).

### Minocycline reduces spatial learning and memory impairment caused by hypoglycemia

Rats were subjected to memory testing using spatial learning in the Barnes maze, a test that relies heavily on hippocampal function. Over the five days of training, all groups learned the spatial task as evidenced by a progressive reduction in the distance traveled to reach the escape tunnel in the Barnes maze (F_4, 200_ = 76.1, *P <* 0.0001). Two-way RANOVA revealed a significant effect of hypoglycemia on spatial learning during Barnes maze acquisition (hypoglycemia effect: F_1,50_ = 5.98, *P <* 0.05). Although minocycline did not have an overall effect on learning (minocycline effect: F_1,50_ = 1.21, *P* > 0.27), it was able to lessen the deficit observed in the hypoglycemic (*P <* 0.05, post hoc test) but not in sham animals (*P* > 0.1, post hoc test). Due to the differential effect of minocycline on euglycemic and hypoglycemic animals, there was a significant minocycline-hypoglycemia interaction (F_1,50_ = 7.24, *P <* 0.01) (Figure 6C).

### Minocycline has a long-term protective effect on neuronal death after hypoglycemia

**Figure 7:**
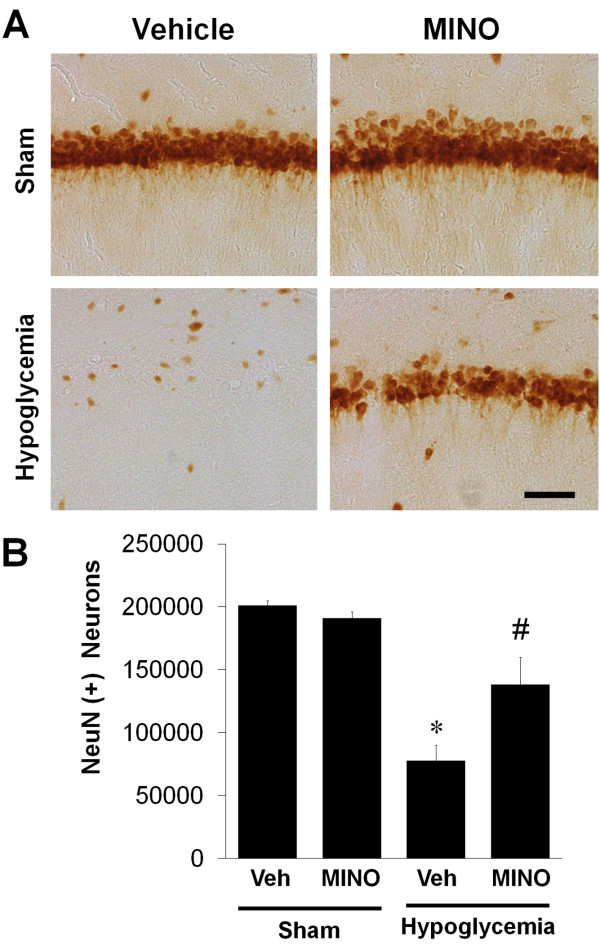
**Delayed treatment with minocycline has long-term protective effects after hypoglycemia. (A)** Representative images of surviving neurons in the post-hypoglycemic hippocampal CA1 area following minocycline treatment. Note that hypoglycemia-induced hippocampal CA1 neuronal death was protected by the delayed minocycline treatment 8 weeks after hypoglycemia. Scale bar = 80 μm. **(B)** Quantification of surviving neurons in the hippocampal CA1 area of rats treated with minocycline (MINO) or saline (Veh) 8 weeks after hypoglycemia. The number of neurons was counted using the stereology method as described in Methods and Materials. Data are mean ± standard error of the mean (SEM); *P < 0.05 compared with sham-operated animals treated with saline (Veh), ^#^*P <* 0.05 compared with hypoglycemia animals treated with saline (n = 6).

To test whether the neuroprotective effect of minocycline on hypoglycemia-induced neuronal death persists for at least 8 weeks post-treatment, we killed animals after behavioral tests and performed histochemistry to assay neuronal viability. At 8 weeks, hippocampus that had undergone hypoglycemic injury showed a loss of neuronal bodies in CA1 area, but minocycline-treated animals had more intact neuronal bodies in CA1 than vehicle-treated ones (Figure 7).

## Discussion

The present study shows that delayed treatment with minocycline reduces microglial activation and neuronal death in the hippocampus after hypoglycemia. Minocycline treatment also prevented cognitive impairment at later time points. These results suggest that minocycline is an effective therapeutic candidate to prevent neuronal death and subsequent cognitive impairment after hypoglycemia.

The mechanisms of hypoglycemia-induced neuronal injury represent a more complex process than a simple lack of glucose supply to the brain [32,33]. Rather, several contributing factors are involved in downstream events of hypoglycemia-induced neuronal death such as sustained activation of glutamate receptors [10], poly(ADP-ribose) polymerase activation [26], zinc translocation [11] and NADPH oxidase activation [12]. Although several studies have been reported to intervene in this neuronal death cascade, there is currently no clinically applicable intervention strategy available. Therefore, this study has sought an indirect intervention strategy for preventing hypoglycemia-induced neuronal death and cognitive impairment that may be more clinically relevant.

The brain injury produced by hypoglycemia maturates over a period of several hours or days as seen in ischemia [34]. Especially in hypoglycemia, delayed hippocampal damage is observed 3 to 5 days after the insult in CA1 pyramidal neurons [35], suggesting that mechanisms that develop slowly after hypoglycemia have a role in hypoglycemic neuronal death. Recent studies have shown that inflammatory cells infiltrate the ischemic area [36] or the hypoglycemic brain area [18]. Inflammation is now recognized as a significant contributing mechanism in cerebral ischemia because anti-inflammatory compounds or inhibitors of iNOS and cyclooxygenase-2 reduce ischemic damage and improve the outcome of animals after ischemic insult [37,38].

Microglial activation contributes to ischemia and traumatic brain injury-induced neuronal death. Previously our lab showed microglia are activated after hypoglycemia [39]. Microglia are the major innate immune cells resident in the brain. Once activated by neurological damage or systemic inflammatory events, microglia release neurotoxic substances such as nitric oxide, ROS, cytokines, and chemokines, and undergo morphological change from a ramified to an amoeboid shape [13,14]. Whether microglial activation is a net harmful or beneficial process is still controversial [15], however, there is evidence that indicates that early phage inflammation by acute brain injury can contribute to neuronal death. Our previous study shows that hypoglycemia induces microglial activation in the brain, which is affected by body temperature and vesicular zinc release [18]. However, it is unknown whether the prevention of microglial activation by minocycline after hypoglycemia is neuroprotective.

Infiltrating peripheral inflammatory cells play important roles in the development of pathophysiological response following neurological damage. Brain damage, such as ischemic or traumatic injury, triggers physiological changes and neuronal death that induces adhesion of circulating leukocytes, leading to their migration into brain, causing release of pro-inflammatory substances [17,40]. MPO produced from neutrophils has been used as a marker of infiltrating neutrophils and is involved in brain damage following such events as traumatic brain injury and cerebral ischemia. Numerous studies have been reported in which the accumulation of MPO-positive neutrophils into the ischemic brain is correlated with ischemic brain damage, although MPO gene deletion has been shown to exacerbate brain injury, which is mediated by the peroxynitrite reaction but not MPO [31,41]. Activation of microglia precedes neutrophil infiltration and seems to play a role in the recruitment of neutrophils following brain damage [42]. In our study, the MPO-positive neutrophils were observed in the hippocampal formation following hypoglycemic brain injury and this recruitment was prevented by minocycline. It suggests that neutrophil infiltration may be involved in brain inflammation and neuronal death after hypoglycemic injury and may be prevented by minocycline treatment.

Although minocycline was developed as an anti-microbial drug for the treatment of various infectious diseases, many other functions such as anti-apoptotic and anti-inflammatory effects have been identified [43]. In particular the anti-inflammatory properties of minocycline have been observed in acute and chronic neurological disease animal models, as well as in human clinical trial studies [22,23,44]. In an animal model of multiple sclerosis, minocycline decreased the transmigration of T lymphocytes and inhibited the activation of metalloproteinases that degrade the extracellular matrix proteins of the basal lamina surrounding blood vessels, causing neutrophil infiltration [45,46]. Based on these studies, our present results suggest that delayed treatment with minocycline can have a neuroprotective effect on hypoglycemic neuronal death by inhibiting microglial activation and neutrophil infiltration.

Since most hypoglycemic patients visit the emergency room several hours after the hypoglycemic episode, we delivered the initial dose of minocycline 6 hours after hypoglycemic insult. Microglial activation was detected in the hippocampus 24 hours after hypoglycemia. Thus we believe that treatment of 6-hours post-hypogecemic insult is a reasonable therapeutic window. Although we injected minocycline from 6 hours after hypoglycemia, microglial activation was significantly reduced, as was neuronal death. These results suggest that delayed treatment with minocycline suppressed microglial activation, which may decrease release of toxic substances such as nitric oxide, and IL6, etcetera. This further suggests that acute microglial activation after hypoglycemia is detrimental to neuronal survival.

Since minocycline is one of the most lipid-soluble tetracycline-class antibiotics, it can easily penetrate into the brain. Minocycline also has a long half-life compared to other tetracycline antibiotics [47]. Thus, one or two doses of 50 to 100 mg per day of minocycline are effective in many patients to treat bacterial infection. A recent clinical study found that patients who received 200 mg of minocycline for five days within 24 hours after ischemic stroke showed significantly better outcome compared with patients receiving placebo [48]. In the present study, we used 50 mg/kg per day for one week in rats. We understand that this concentration of minocycline is fairly high for a single dose. Therefore, for clinical applications, a single dose of minocycline for prevention of hypoglycemia-induced neuronal death should be re-evaluated.

Learning and memory deficits are common neurological sequelae following hypoglycemia in patients with type 1 diabetes and in the relatively younger population with type 2 diabetes [49-51]. Our previous study showed that hypoglycemia-induced hippocampal damage induced impairment of learning and memory [26]. In the present study, the Barnes maze test was performed to evaluate spatial learning and memory. As seen in our previous study using the water maze test, subjects experiencing severe hypoglycemia displayed a longer distance traveled to reach the escape tunnel, implying that spatial learning has been compromised. However, minocycline treatment reduced the travel distance in rats who experienced hypoglycemia. It has been reported that minocycline improves cognitive impairment in focal cerebral ischemia [52], Alzheimer’s disease models [53], and other animal models of neurological disease [54,55].

Because tetracycline derivatives, like minocycline, have anti-inflammatory properties and are clinically well tolerated, we studied whether minocycline could serve as a neuroprotective compound against hypoglycemia-induced brain injury. In the present study, we report that in a rat model of hypoglycemia, 1) minocycline is neuroprotective, even when the treatment is initiated 6 hours after hypoglycemia; 2) minocycline prevents microglial activation after hypoglycemia; and 3) minocycline prevents cognitive impairment even at several weeks after hypoglycemia. Thus, the present study suggests that prevention of microglial activation by minocycline has a strong therapeutic potential for prevention of hypoglycemia-induced brain injury.

## Competing interest

The authors declare no competing interests.

## Authors’ contributions

SW, JK, BY, MP, HK and TK researched the data. MS performed the data analysis. SW, JL, SS reviewed and edited the manuscript. SW, JL and SS wrote the manuscript and contributed to the discussion. All authors have read and approved the final manuscript.
